# Prevalence of Sexual Violence in Migrants, Applicants for International Protection, and Refugees in Europe: A Critical Interpretive Synthesis of the Evidence

**DOI:** 10.3390/ijerph15091979

**Published:** 2018-09-11

**Authors:** Lotte De Schrijver, Tom Vander Beken, Barbara Krahé, Ines Keygnaert

**Affiliations:** 1 UGent-International Centre for Reproductive Health, 9000 Ghent, Belgium; lotte.deschrijver@ugent.be; 2 UGent-Institute for International Research on Criminal Policy, 9000 Ghent, Belgium; tom.vanderbeken@ugent.be; 3 Department of Psychology, University of Potsdam, 14476 Potsdam, Germany; krahe@uni-potsdam.de

**Keywords:** sexual violence, migrants, refugees, asylum seekers, applicants for international protection, Europe, prevalence

## Abstract

*(1) Background*: Sexual violence (SV) is a major public health problem, with negative socio-economic, physical, mental, sexual, and reproductive health consequences. Migrants, applicants for international protection, and refugees (MARs) are vulnerable to SV. Since many European countries are seeing high migratory pressure, the development of prevention strategies and care paths focusing on victimised MARs is highly needed. To this end, this study reviews evidence on the prevalence of SV among MAR groups in Europe and the challenges encountered in research on this topic. *(2) Methods*: A critical interpretive synthesis of 25 peer-reviewed academic studies and 22 relevant grey literature documents was conducted based on a socio-ecological model. *(3) Results*: Evidence shows that SV is highly frequent in MARs in Europe, yet comparison with other groups is still difficult. Methodologically and ethically sound representative studies comparing between populations are still lacking. Challenges in researching SV in MARs are located at the intrapersonal, interpersonal, community, societal, and policy levels. *(4) Conclusions*: Future research should start with a clear definition of the concerned population and acts of SV to generate comparable data. Participatory qualitative research approaches could be applied to better grasp the complexity of interplaying determinants of SV in MARs.

## 1. Introduction

Sexual violence (SV) is a major health, judicial, and societal concern [1,2] and can have numerous serious short- and long-term physical, psychological, and social consequences for victims, but also for family members, peers, and assailants [1,3,4,5]. It is a global and serious public health and human rights problem [5]. SV occurs all over the world, in all cultures, at every societal level, among people from all genders, and in all age categories [1]. Regardless of the context in which SV occurs (during war and conflict, within an intimate partnership or larger family or community structure), it is considered a deeply violating and painful experience for those affected [5]. SV can be broadly defined as a range of behaviours including sexual harassment, sexual violence without penetration, and attempted and completed rape, and can occur in a myriad of contexts and relationships [6]. It includes victimization, perpetration, and the witnessing of transgressive and violent sexual acts, taking place between strangers or in close and intimate relationships, motivated by individual or political reasons, in the context of conflict, exploitation, or targeting of a specific group [7]. In this paper, we will use the term “SV” to refer to this broad range of behaviours. However, since there is a diversity in the use of terms referring to SV, we will use the terminology of the original papers in the “results” section in order to be clear about the specific types or subcategories of SV addressed in the respective studies.

Both scientific and grey literature identify applicants for international protection and refugees as being especially vulnerable to SV exposure [8,9]. However, the extent to which this vulnerability is reflected in the prevalence of SV is not yet made clear. There is a lack of qualitative and comparable research on this topic. Moreover, the implementation of prevention and response policies based on the evidence is rare.

In the last decade, the world has seen an increase in the number of people forced to flee from their homes. In 2014, the United Nations High Commissioner for Refugees (UNHCR), counted 59.5 million forcibly displaced people worldwide, which was a 16% increase compared to the previous year [10]. The numbers have gone up since then. Today, the UN Agency estimates that 65.6 million men, women, and children have been forced to leave their home countries. This figure includes 22.5 million refugees, of whom over half are minors under the age of 18 [11].

The terms “migrant”, “asylum seeker/applicant for international protection”, and “refugee” are often considered as synonyms but refer to different populations. In this paper, the definitions proposed by the UNHCR will be used. A migrant is someone who consciously and voluntarily decides to leave his/her country of origin and who could decide to go back without having to fear for their safety. Others who leave their home country do not have that option [12]. Asylum seekers or applicants for international protection flee their home country and are awaiting a decision on their request for international protection [12]. Refugees are those applicants that have received a positive decision regarding their request for international protection. The abbreviation “MARs” will be used here to refer to migrants, applicants for international protection, and refugees. Although the focus of this study is mainly on the latter two, the term “migrant” is also included, because a migrant may have been an applicant or refugee in the past.

Although the number of applications for international protection has recently declined, applicants and refugees still remain a group not to be neglected [13]. Although Europe has a long history as an area for migratory transit and/or as a destination, the high numbers of MARs have provided some challenges. One of them is the responsibility to guard, guarantee, or aid in achieving the health and well-being of MARs.

To take up this responsibility, a clear overview of the specificities regarding the population’s experienced threats to health and well-being should be identified in order to develop and provide adequate and appropriate care. Since the harsh conditions in which MARs may find themselves may hamper their access to protection and medical, forensic, and/or psychological care in European countries, it is essential to research the impact of SV on the lives of those belonging to vulnerable groups. This will contribute to a better understanding of the mechanisms involved and to recommendations with an eye to improving the health and wellbeing of all individuals belonging to this group.

A first step in the research chain is to estimate the magnitude of the problem in this population. The objective of our study is to critically review the evidence on the prevalence of SV in MARs in Europe and to discuss the challenges of conducting research on SV in this specific population to inform future prevalence studies. To address the study objectives, this review will look into prevalence studies on SV in MARs in Europe through the method of a critical interpretive synthesis (CIS).

## 2. Methods

### 2.1. Critical Interpretive Synthesis

In order to critically discuss the evidence and challenges of research on SV prevalence in MAR, we opted to conduct a critical interpretive synthesis (CIS). This method not only uses the conventional search process of traditional systematic reviews, but focusses on identifying and selecting a diverse sample of documents [14] stemming from both academic and policy as well as legal frameworks. A CIS thus also considers studies that would not meet the criteria for inclusion in a systematic review, in particular grey literature including research reports from non-governmental organisations (NGOs) working directly with the population, for example. This broadens the scope significantly and helps us to better grasp the heterogeneous body of literature on SV in MARs [15].

The strength of a CIS lies in promoting an understanding of phenomena and theory construction through an inductive and interpretive combination of constructs and evidence from different approaches and study fields, resulting in a new coherent whole [16]. This methodology allows us to integrate different kinds of sources, regardless of whether they are peer reviewed or grey literature, and to add a qualitative dimension to the analysis [16].

Different from traditional systematic review methodologies, a CIS does not draw upon an a priori defined research question representing a specific hypothesis, but rather identifies and refines relevant questions during the review process itself [17]. Its process is characterised by a dynamic, interactive, and recursive nature with recognition of the necessity of flexibility and reflexivity during the search, sampling, and analysis of the data [16,18]. Instead of a linear progression, the CIS review strategy is more circular and evolves organically, explicitly acknowledging that some aspects of the process will not be auditable or reproducible as it is grounded in the evidence retrieved by specific research teams with their particular backgrounds and awareness of relevant literature from various fields and sources [16,18]. The non-linear progress of this method also allows for including new sources based on the findings of the interpretive synthesis and to analyse the data in the light of different research areas and specific background knowledge of the research team, which may ultimately contribute to the construction of new theoretical frameworks [16]. New theories and concepts arising as a result of a CIS may serve as a starting point for future studies [16].

To answer the predetermined research objectives, we synthesized and approached the literature through the lens of a socio-ecological model. This approach [19,20] can be used to study SV from a perspective in which four interlinked levels play a key role. It suggests that the interactions between the individuals and their environment shape their development over time [19]. Within a complex system of relationships affected by multiple levels of the surrounding environment [20], research on SV takes place in a social context. These interactions between different social contexts can be categorized in various systems.

At the first level of the socio-ecological model, we find the intrapersonal processes taking place within the individuals themselves. These internal processes are bi-directionally influenced by the immediate surrounding of the individual, the second level. The relationship between the individuals and their direct context is, in turn, affected by the relationships between several different microsystems within a community, representing the third level. At the fourth level, communities are influenced by processes taking place at the societal and public policy levels [19,20].

Starting from this model, we begin by looking at relevant factors in the light of SV prevalence in MARs identified at the individual level, followed by challenges at the interpersonal level, in order to continue with the organisational and community level and finally the societal and public policy level.

The CIS method combined with a socio-ecological approach guides us in considering why little is known about the prevalence of SV in Europe, how the identified research challenges relate to each other [18], and how they may influence recommendations for practice [17] and future research. Although papers on related topics have been published in the past, these mainly concerned systematic reviews and did not include relevant non-academic sources. The approach we used here allows us to add to the knowledge base on this topic by critically evaluating on which socio-ecological levels researchers and policymakers should focus in order to improve research strategies and policies concerning SV in MARs in Europe.

### 2.2. Sample of Studies

Between 1 May and 31 August 2017, we selected peer-reviewed articles relevant to the research objectives of this study through a database search. During the first phase of the CIS, articles were deemed relevant if they contributed original data, theoretical or methodological information, or policy considerations to the problem of SV in MARs living in Europe, regardless of the specific research question, the study field, or research design. This search was part of a larger study called “UN-MENAMAIS-UNderstanding the MEchanisms, NAture, MAgnitude and Impact of Sexual violence in Belgium” and took place simultaneously with the search action for a CIS on the risk factors and consequences of SV and help-seeking behaviours after SV victimization in MARs. These results will be presented elsewhere.

The following inclusion criteria were used: papers on SV in MARs in Europe had to be published after the year 2000 in English, French, or Dutch. Any author’s definition of sexual violence (i.e., rape and any other form of sexual violence) was accepted, but studies that combined sexual and non-sexual violence into one category in the analysis were excluded in order to maximize the comparability of the prevalence numbers. However, we did use these sources in order to obtain a deeper sense of the background concerning the issue of SV in MARs. If different forms of violence were studied in one single study and rates of SV could be separated from the other types of violence, the paper was still included. The Medical Subject Heading (MeSH, which refers to the controlled vocabulary used to index all of the articles in PubMed) terms and key words presented in Table 1 were used in different combinations and databases.

In addition to peer-reviewed articles, we also considered grey literature. With the term “grey literature”, we refer to those documents that have not necessarily undergone a peer-review process and/or are not included in academic bibliographical retrieval systems and often remain unpublished and limited in distribution [21,22]. It includes reports of different natures, including dissertations, technical specifications, standards and guidelines, official documentation, and so on [21]. These sources were derived from the reference lists of studies used in the first phase and sources provided by experts and alerts.

Initially, we identified a total of 2380 peer-reviewed articles on the prevalence of SV, its consequences, the associated risk factors, and help-seeking behaviour after SV in MARs in the database search and 93 through other sources. Of those, 138 documents were selected as relevant for the general CIS on SV in MARs in Europe (cf. above). Ninety-one documents were excluded from the CIS because they did not address our research question on SV prevalence in the population of interest as they were of poor quality or were not fully accessible for our research team. We ultimately included 25 peer-reviewed articles and 22 grey literature documents in the CIS on the results and challenges of SV prevalence studies in MARs in Europe. To have an idea of the prevalence of SV in MARs in Europe, only papers addressing the problem of SV in MARs living in Europe were included in the analyses. The studies retained in the CIS discuss SV prevalence in MARs in the following 12 European countries: Belgium, France, Germany, Greece, Hungary, Ireland, Malta, the Netherlands, Portugal, Spain, Sweden, Switzerland, Turkey and the United Kingdom. One study reported a survey conducted in all EU countries.

## 3. Results

### 3.1. Prevalence of SV in Migrants, Applicants for International Protection, and Refugees in Europe

Compared to the attention given to SV in the general population, research on SV in MARs is extremely scarce. In this section, we discuss the prevalence of SV victimization and perpetration in MARs in Europe. The findings regarding both victimization and perpetration will be presented together when they stem from the same studies.

As we will discuss in more detail later, estimating the prevalence of SV in MARs involves several challenges. The combination of these challenges led to the identification of only five studies on the prevalence of SV in MARs in Europe that matched our predetermined inclusion criteria, and may be the primary reason for the scarcity of data on prevalence, consequences, and mediating factors of SV in this population.

One frequently cited paper concerns a community-based participatory study by Keygnaert et al. from 2012 [23] on sexual and gender-based violence (SGBV) in refugees, applicants, and undocumented migrants in Belgium and the Netherlands. This study showed that approximately 57% of the participants indicated that they had been confronted with SV experiences, comprising rape and sexual exploitation among others. Compared to the general population, the nature of the experienced SV included more incidences of multiple and gang rape [23]. A fifth of all respondents in this study reported having been sexually victimised themselves. A total of 332 acts of SGVB were described in 223 interviews, including 188 cases of SV, of which 47 were personal experiences and 141 were experienced by a close peer of the respondent [23]. These results indicate a high risk for MARs of becoming confronted with SV. At a more detailed level, 69% of individuals victimized since their arrival in Europe were women, and 29% were men. In 2% of the cases, the gender of the victim remained unclear or multiple victims of both sexes were involved. The opposite pattern can be found when asking about who the assailants were. In 72.6% of the cases, the assailant was a man, in 1.5% it concerned a transgender assailant, in 19.6%, the gender was not clearly specified, and only in 6% of the cases the assailant was a woman [23]. In line with findings from studies in the general population [4,24], most victims knew their assailants [23]. The assailant was in most cases an intimate partner (31%) and less frequently a professional (23%), a family member (16%), an acquaintance (15%), or a stranger (12%) [23]. In a third of the incidences, the assailant was a European citizen [23].

When looking further into the European situation, another much-cited study by Keygnaert et al. [25] from 2014 in eight European countries (Belgium, Greece, Hungary, Ireland, Malta, the Netherlands, Portugal, and Spain) examined the prevalence of SV in refugees, applicants for international protection, and undocumented migrants in a wider European context. This community-based participatory study showed that in the European asylum reception sector, both sexes as well as both residents and professionals are at risk of being exposed to different forms of violence, including SV, with 58.3% of the 562 respondents reporting having being directly (23.3%) or indirectly (76.6%) confronted with SV [25]. This study also showed that victimization and perpetration of violence seem to be more gender-balanced in comparison with the general population. Both sexes indicated a comparable tendency to have experienced all types of violence perpetration and victimization. However, men were more likely to be involved in SV perpetration and emotional victimization, whereas women tended to be more likely to become the victim of SV and perpetrate emotional violence [25].

It should be noted here that the findings may be an underestimation since many of the respondents in the study indicated that they did not want to disclose or were hesitant to disclose personal SV experiences as long as they remained and/or worked in reception centres. Fear of reprisals by community members and feared impact on their asylum case or stay in the facility may have influenced the number of reported cases [25]. Although these studies give us an idea of the magnitude of the problem in this population, their objective was not to present representative prevalence numbers.

In the same period, the European Union Agency for Fundamental Rights (FRA) [9] published an EU-wide survey about violence against women (VAW), which included a section on the prevalence of violence in migrant populations. They concluded that women who were not a citizen of their current country of residence were more likely than women without a migration history to become victims of physical and/or SV by both partners and non-partners after the age of 15. However, they did not find notable differences from the general population in physical, sexual, or psychological violence before the age of 15, and sexual harassment and stalking after the age of 15 [9]. Unfortunately, these numbers cannot be broken down according to legal status, do not consider SV against men, and are not comparable with the figures by Keygnaert et al. in 2012 and 2014 [23,25].

In 2015, Doctors of the World (*Médicins du Monde*, MdM) [26] disseminated a report on the access to healthcare in 2014 for vulnerable people, such as MARs in 11 countries (Belgium, France, Germany, Greece, the Netherlands, Spain, Sweden, Switzerland, the United Kingdom, Turkey, and Canada). The data were collected by means of social and medical questionnaires administered to patients who attended a MdM consultation in one of the 11 countries [26]. Unfortunately, questions on SV were not asked in every country and strongly depended on the healthcare providers’ willingness to address the issue. Even though the numbers could not be generalized, the results indicate that applicants for international protection were disproportionately highly represented among victims of violence [26] and thus may support the hypothesis that MARs are more vulnerable to SV than the general population. Among the patients who were questioned about their experiences with SV, 27.6% (37.6% of women and 7.3% of men) reported sexual assault. Rape was mentioned by 14.9% of those patients (24.1% of the women and 5.4% of the men). Interestingly, male patients reported a quarter of the total number of sexual assaults [26]. The figures cannot be considered representative, but they provide an impression of the current situation.

Another important finding in this study, which continues in the line of the results found by Keygnaert et al. [23,25,27], relates to the experiences of SV throughout the trajectory of MARs from country of origin to country of destination. MARs do not only experience SV before migrating, but also during and after their arrival in Europe [26,27]. In the population studied by MdM, 21.1% of the reported rapes and 17.7% of the sexual assaults took place after the victim’s arrival in the host country. This is an important finding since most studies on SV in MARs only consider violence cases in the country of origin, ignoring the experiences en route or after arrival. Keygnaert et al. [27] found in another study published in 2014 on SV among sub-Saharan migrants in Morocco that 45% of them had experienced SV in a direct or indirect manner during their migration or in Morocco itself. These are important findings to consider when asking about SV in this population. Identifying aspects related to SV before leaving the country of origin, during transit, and after arrival in the host country is necessary in order to provide appropriate and adequate policy recommendations and prevention strategies.

In 2009, the Refugee Council’s Vulnerable Women’s Project (VWP) [28] published some numbers in line with the trends reported here. In a 21-month period (2006–2008), the project supported 153 refugee and asylum-seeking women in the United Kingdom. Of those, 76% indicated that they had been raped either in their home country or in the United Kingdom, had been sexually abused (22%), or had been confronted with threats of being raped or sexually abused while in detention in their country of origin (9%). Men were not included in this study [28].

We will briefly discuss some non-European studies on conflict-related SV (CRSV) to illustrate the specific SV experiences that may have taken place before the migration process started. During the Rwandan genocide for example, up to half a million women were raped. In parts of Liberia, more than 90% of women and girls above the age of three became the victim of CRSV, and in parts of Eastern Congo, it is estimated that about 75% of the women were confronted with SV [28]. CRSV presents itself in specific forms, such as gang rape, depending on the war in which it takes place and the underlying function of the practice [29,30,31,32].

Although clear and robust prevalence rates of SV in MARs in Europe are lacking, the evidence we have right now supports the hypothesis that MARs are vulnerable to becoming victims of SV. In addition, it is in line with the wide recognition that in times of conflict everyone is more exposed to violence and more particularity to SGBV [33]. The numbers reported on the situation in Europe also follow the same trend as shown in a systematic review and meta-analysis from 2014 on SV among female refugees in complex humanitarian emergencies. Vu et al. [34] estimate the prevalence of SV in this population as approximately one in five women. Given the multiple barriers associated with disclosing the experiences, the researchers stress that these numbers are most likely an underestimation.

### 3.2. Challenges in Conducting, Comparing, and Interpreting Research in Migrants, Applicants for International Protection, and Refugees

The challenges in conducting research on MARs were approached and analysed in this study from a socio-ecological perspective [19,20] in which four interlinked levels play a key role. We start by (1) looking at the issues identified on the individual level, and continue with (2) the interpersonal level, followed by (3) challenges at a broader organisational and community level. We will finish this analysis with (4) a discussion of barriers in conducting research at a societal and public policy level.

#### 3.2.1. Research Challenges at the Individual Level

At the individual level, two perspectives need to be taken into account, namely that of the researcher and that of the research participant. Given the experience of the MAR population with violent conflict, displacement, and human rights violations, most researchers struggle with approaching their study population purely as objects of research without trying to make a difference and reduce suffering [35,36]. From the researchers’ point of view, remaining neutral can be quite a challenge. In addition, from an ethical perspective, one may question the motives for researching MARs if researchers cannot offer them anything in return.

From the point of view of the research participant, factors related to disclosing sensitive information about themselves can be a perceived or real threat to personal safety (infra). This may result in (dis)trust from migrants of the so-called authorities and may influence their willingness to participate.

#### 3.2.2. Research Challenges at the Interpersonal Level

Many researchers are intrinsically motivated to work with this population because they want to contribute to improving their situation [35]. At the same time, research can only have a substantial impact for a group of people if the results are published. Both ethical considerations and logistic challenges are central to finding the balance between applying high academic standards to the research design and making a difference to the participants. It is not always possible for researchers to reveal the details of how they conducted the study (e.g., identification and selection of participants, handling of local security issues, context of the interviews, access to illegal immigrants, illegal activities performed by MARs, etc.), because of the privacy of research participants and the fact that their safety might be at stake. The political and legal issues related to the situation of applicants for international protection and refugees means that they have fewer rights and are at risk when participating in research [35,36]. Because of the protection of the safety of the participants, some elements of the research design may not always be revealed. The manner of gaining access to undocumented migrants is one example. Participating in research on illegal behaviour may put both respondents and researchers at risk [36]. Withholding concrete and detailed descriptions of the research process and the gatekeepers involved as a way of protecting individual participants and the community as a whole [36] may lead to a lack of reproducibility of the study and threaten the transparency of the results.

Researchers and their respondents often do not speak the same language and might have different cultural backgrounds [35], meaning that interpreters or cultural mediators have to be involved in order to gather data. However, including a third party into qualitative research brings along new challenges, such as erroneous translations, difficulties in establishing a relation of trust between the interviewer and the interviewee, and the risk of interviewees refraining from disclosure in the presence of a third party out of fear of the effects on the community to which they belong.

These challenges could be reduced through the use of a community-based participatory research approach [6,37]. This approach creates bridges between scientists and communities and establishes mutual trust by sharing knowledge and valuable experiences [6]. By participating within the community, researchers gain a deeper understanding of the unique circumstances in which a given community lives. It facilitates open dialogue on sensitive issues and helps to define mutual agreements about the collaborative research process [6].

#### 3.2.3. Research Challenges at the Organisational and Community Level

Another significant challenge relates to the fact that the research population of MARs is mobile and hard to reach. Firstly, when the study population is characterised by being on the move, it is difficult to investigate effects over longer periods of time. This is specifically relevant in the light of the impact of SV on the lives of victims, assailants, and their families/peers. Longitudinal designs are very hard to establish.

Another fundamental problem in conducting representative studies on all kinds of migrant populations lies in the difficulties of getting access to a certain community. To start with, MARs are not equally distributed over Europe, nor within each country. They often live in big cities or near a certain reception centre, and even within these broader areas they tend to live in specific neighbourhoods. This means that achieving nationwide randomized samples is very costly and resource-intensive [38,39]. Furthermore, due to their legal status (or rather the lack thereof), subpopulations of MARs often remain hidden [38,39]. The combination of these factors makes MARs a population that is hard to reach.

#### 3.2.4. Research Challenges at the Societal and Public Policy Level

The societal level in the socio-ecological model looks at the broad societal factors that create a challenging climate to conduct research on a vulnerable population such as MAR. First of all, regulations regarding legal statuses can significantly influence research opportunities. Ethical considerations play an important role here. Is it ethical to ask research questions which may ultimately lead to policies that may negatively impact the living situation of the studied community? Düvell et al. [36] gave the example of documenting *how* undocumented migrants enter a country compared to studying the *why* question with regard to this behaviour. Researchers’ findings may be used by policymakers to the disadvantage of the communities that participate [36]. A thorough reflection of the justification of why one wants to conduct a certain study and how the findings may be used afterwards should always be part of the preparatory phase.

Secondly, the extent to which people with different legal statuses are integrated into the larger administrative and demographic organisation of a society can have a strong impact too. A common practice to register migrants in the European Union is missing [38,39]. A related obstacle concerns the difficulty of including MARs in representative studies. MARs are often excluded from large national studies because of language problems [24] and a lack of complete demographic information [38,39]. Undocumented migrants also remain excluded from these studies since they are not represented in national registries [38,39].

### 3.3. Challenges in Conducting Research on Sexual Violence

In addition to dealing with the specific challenges involved in studying MARs, research on SV itself can equally be a challenging task. Again, several reasons for the lack of an extensive, systematic knowledge base on this topic can be classified according to a socio-ecological framework.

#### 3.3.1. Research Challenges at the Individual Level

The primary reason for the difficulty of establishing the magnitude of the problem lies in the fact that those involved commonly hide the experience (infra) and health care workers do not recognize it. This may be out of fear of being stigmatized or of further violence after disclosure [24,28,40,41]. One cannot count or study what remains hidden.

#### 3.3.2. Research Challenges at the Organisational, Community, and Interpersonal Level

The organisation and structure within the community and institutions create a specific barrier in gaining access to populations. The refusal by community gatekeepers or those in charge of institutions reduces the access to individuals that may be relevant for researchers [6,38,39]. This leads to bias and a lack of generalizable data.

Linked to gaining access to a certain community to talk about SV are interpersonal barriers related to the sensitivity of the researched topic. Disclosure of SV is one of the most important interpersonal challenges identified with regard to investigating SV. Respondents need to be actively motivated to discuss their experiences with SV to provide researchers with the necessary details to arrive at conclusions that correspond with the lived reality of the participants. Therefore, certain criteria should be met. Participants will be more likely to talk about their experiences if they perceive the interviewer as trustworthy, as someone who understands them, as someone who responds in an accepting and not stigmatizing manner, and if they expect disclosure to lead to future benefits [42,43,44]. These benefits could be personal, but could also be related, for example, to the prevention of future victimization of others. Drawing the bigger picture of a study in that light could be very useful. Informing participants about what will ultimately happen with the findings of the study may be one way to increase the motivation for participation. Another strategy may be to involve them in the dissemination of the results within the community and to policymakers afterwards [2]. The use of a participatory research design could thus be a promising approach to incentivise engagement in SV research.

#### 3.3.3. Research Challenges at the Societal and Public Policy Level

At a broader societal level, it appears that defining SV remains a significant challenge. What falls under the category of SV is not always clear. Publications on the theme use different terms to describe the same concepts and phenomena or describe different types of SV with the same terminology. Further, the societal construction and awareness of SV often seems to be limited to female victims of rape perpetrated by men [6,45,46]. The victimization of males and transgender people is generally neglected [45,46]. Stereotypical thinking about victims and sexual violence is strongly reinforced in ruling rape myths [47]. As we will discuss below, this impacts the policies and funding of studies in specific populations and the focus of research questions.

##### Definitions of Sexual Violence

Defining SV in a consistent manner is an important issue in researching and reporting SV to avoid confusion and enhance the comparability of findings [48]. In a number of studies for example, data on both sexual and physical violence are collected and/or analysed as one single item. When presented as a single item, the nature of the violence and the underlying dynamics remain unclear. Both physical and sexual violence encompass a multitude of types of violent acts, may emerge in diverse contexts, and result in different consequences. At the same time, physical and SV show some overlap, are often linked to each other, and may be hard to distinguish from one another in certain situations [6,49]. Specifically, in the light of intimate partner violence (IPV), the distinction between the two may be unclear. SV encompasses all sexual acts against someone’s will. It may be difficult to judge whether a sexual act in an intimate relationship is against a partner’s will. For example, when partners have sex with their partner against their will to avoid physical violence, does this count as SV or not? A clear distinction between the two is often not described, and definitions are lacking.

The lack of a clear and encompassing definition of SV may result in policies and research funding that focus solely on those forms of SV (such as completed rape of women) that meet the lay understanding of the general public rather than corresponding to how the phenomenon is really perceived and experienced by those people affected by it [6].

##### Violence against Women and Gender-Based Violence (GBV) as Umbrella Terms

The same problem arises in the study of VAW or GBV. SV is often discussed under those broader umbrellas that are often used interchangeably [28]. GBV is generally used to describe and capture all forms of violence that occur as a result of “the normative role expectations associated with each gender, along with the unequal power relationships between men and women within the context of a specific society” [50] (p 14).

VAW could be considered as a subcategory of GBV in which violence is directed at girls and women and goes beyond what we consider as SV alone. It refers to many forms of violence, including IPV and rape/sexual assault and other forms of SV perpetrated by someone other than a partner (non-partner sexual violence), as well as female genital mutilation (FGM), honour killings, and the trafficking of women [4]. Note that while women, girls, men, and boys can all become victims of GBV [23,27,51], the main focus within this research area has traditionally been on women and girls. To illustrate, an estimated 35% of all women worldwide are confronted at least once during their life with physical and/or sexual violence, IPV, or SV by a non-partner [4]. When it comes to lifetime prevalence in men however, these numbers are not available.

From a gender perspective, as a result of a ruling patriarchy, power relations, and hierarchical constructions of masculinity and femininity, women appear to be more vulnerable to structural gender inequality [24,52]. Therefore, the primary focus within GBV lies on VAW [24], which creates a gap in the knowledge about GBV against boys and men [1,51] and undermines the comparison of female and male experiences of victimization.

##### Defining Human Trafficking and Sexual Exploitation

Other umbrella terms related to SV and often used in the context of MARs are “human trafficking”, “sexual exploitation”, and “SV as a weapon of war or conflict-related SV” (CRSV). These types of violence could play a role both in the decision to leave the home country or in the specific vulnerabilities of MARs while in transit or after arrival in the host country in Europe.

*Trafficking in persons* or *human trafficking* are often used as synonyms. Trafficking is characterized by the exploitation of vulnerable people in specific kinds of way. The United Nations Office on Drugs and Crime (UNODC) defines it on their webpage as “recruitment, transportation, transfer, harbouring or receipt of persons, by means of the threat or use of force or other forms of coercion, of abduction, of fraud, of deception, of the abuse of power or of a position of vulnerability or of the giving or receiving of payments or benefits to achieve the consent of a person having control over another person, for the purpose of exploitation” [53]. One way of exploitation is *sexual exploitation*, a form of SV that again covers a range of different forms of sexual violence, such as forced prostitution, sexual slavery, transactional sex, solicitation of transactional sex, and having an exploitative relationship [54,55]. The UN describes it in its glossary as “any actual or attempted abuse of position of vulnerability, differential power or trust, for sexual purposes, including, but not limited to, profiting monetarily, socially or politically from the sexual exploitation of another” [55].

Estimates of trafficking for sexual exploitation are difficult to ascertain for a number of reasons. One of the complexities is the fact that the boundaries of trafficking and exploitation are often hard to define [56,57]. Again, consensus on a clear definition of the practices is lacking. Ascertaining prevalence rates can thus be a challenging task. Nevertheless, there are enough indications of the magnitude of this problem to draw the conclusion that it is a serious issue, especially among MARs. The number of migrant girls and women under sexual exploitation at any given time in the United Kingdom is estimated by the Refugee Council to range between 4000 and 10,000 [28]. A survey from 2012 by the International Labour Organization (ILO) showed that an estimated 22% of people in forced labour were sexually exploited. The organization also estimated that two-thirds of all the revenues from forced labour globally were the result of some form of forced sex work, amounting to around 99 billion US dollars or 85 billion euros a year [58].

As we will discuss later, migrants are especially at risk of ending up in forced labour and experiencing SV in this context. According to the ILO, 44% of the victims had migrated within or across countries prior to being trafficked [58]. The ILO also indicates that the vast majority of victims of sexual exploitation are women and girls [56,57,58]. Importantly, we should not forget that the focus on females as being the only victims might lead to a biased image. Based on different literature reviews, multiple authors concluded that the existence of male sex workers was not acknowledged in the identified sources [59,60].

In those cases where male sex workers were mentioned, they seemed to be considered as less severely victimized. In contrast to female sex workers, they were assigned much more agency. Further, in studies on male victimization, the focus was more on the danger of HIV infection rather than on the violence component [59], emphasizing the public-health threat rather than the need for care [6] of male victims. Providing health care for MARs appears to be motivated primarily by removing a threat to public health [61,62]. In this regard, focusing on infectious diseases is often more accepted than, for example, investing in mental health care, which is often considered as only benefitting the individual involved.

Keygnaert and Guieu [61] argue that the binary approach in SV research, seeing women as victims and men as perpetrators, ignores the complexity and multiplicity of the experience of violence, women’s agency and victimisation of male, lesbian, gay, bisexual and transgender (LGBT) individuals victimization, and the role of reigning social norms leading to acceptance of violence [61]. Interestingly, in studies on male sex workers, sexual orientation seemed to be an important aspect, whereas female victims of sexual exploitation were automatically considered to be heterosexual [59]. SV against men seems to be recognized only when it concerns the rape of male prisoners or sexual torture of homosexual men [41,63].

##### Conflict-Related Sexual Violence (CRSV)

SV may also occur as a component of war and conflict. Situations during or after conflict are contexts with a high prevalence of SV [1]. CRSV refers to a potential weapon of war, ethnic cleansing, or genocide, and is widely acknowledged as a serious problem of international security [30]. This type of SV may be different from other types in that it is used as a strategical means to attain a goal, namely achieving power and dominance over a group considered as inferior [29]. SV probably occurs in all conflicts, but the prevalence and severity differs widely [30,31,64,65]. The term “CRSV” covers all acts of SV that can be considered as strategical mechanisms deployed to attain military or political goals [66]. However, wartime rape is not necessarily always an intentional war strategy, but is often a tolerated weapon rather than an ordered way of attacking the enemy [29]. It is used as a way of torturing people to exercise control over a specific group of people (e.g., ethnic minorities) or as a way to punish or offend individuals and the group to which they belong [28,32].

It is important to consider all actors in conflict situations as possible victims and assailants. Making the distinction between the two roles in conflict areas can be very difficult [32]. Militias, rebels, state officers, and civilians are at risk of becoming victims, assailants, or both in the context of war, with armed state actors being identified as more frequently perpetrating SV than rebel groups [29]. In the literature concerning CRSV, we can again identify a gender bias given that researchers generally do not ask about the sex of the assailants, but assume they are male [29]. Where testimonies about CRSV against men do exist, they are often minimalised, not classified as SV, or ignored. Although the literature is scarce, forced fellatio and masturbation, genital mutilation, forced rape by civilian men, and forced insertion of objects in the anus of prisoners have been described during conflict [41,51,67]. Given this observation, it becomes clear that better knowledge about SV against boys and men is needed.

In combination with the diversity of SV in conflict areas, making statements about the general prevalence of CRSV is again not possible to date. In addition, apart from problems with defining SV from an relational and societal perspective, the terms “rape”, “sexual assault”, “sexual abuse”, and “sexual violence”, which are important to distinguish as they might include or exclude different acts a victim had been subjected to, are often considered to be synonymous and are in many papers used interchangeably [1]. This may result in blurred prevalence numbers.

Due to the lack of clear definitions used in studies, comparable prevalence data on CRSV is difficult to collect.

## 4. Discussion 

By discussing the evidence and challenges of SV prevalence research in MARs in Europe based on a CIS in the light of a socio-ecological model, this paper adds to the limited knowledge base on this topic. It does so by placing the findings from both peer-reviewed studies and grey literature next to each other and by analysing both the specific challenges of conducting SV research and prevalence studies in MAR populations. The findings of this CIS allow us to formulate some recommendations for future research. Firstly, there is a pressing need for high-quality representative prevalence studies on SV in MARs in Europe. Secondly, the identified challenges in conducting research on MARs lead to the conclusion that a clean and ethical design for conducting research within this particular population may be hard to reach and that creative approaches and mixed methods may be necessary. Designing a study with this population would require attention to the specificities of MARs and their situation. This means that we, as researchers, need to look for ways of reaching MARs who could fall out of samples because of their legal status and cultural/language barriers. In addition, we need to guarantee the safety of the participants. This can be done through a thorough analysis of the safety threats on all levels of a socio-ecological approach and through addressing them in an ethical and concrete way before the start of the study. Past researchers have experienced that participants were more willing to talk about the experiences if they were not dependent for care or reception on the facilities in which they were interviewed [23,25]. Considering the dependent situation in which MARs may find themselves is one example of how we can estimate the safety threats for participants in SV research.

By working with interviewers who speak the same language and have the same cultural background as the participants, misinterpretation of the data due to linguistic errors could be avoided. However, researchers should be sensitive to the possible introduction of cultural biases by those interviewers and discuss the interpretations with them. Further, although this might avoid the problem of having to work with interpreters, it might induce a barrier on the side of the respondent as this person might take into consideration the cultural habits of disclosure on such topics and potential harmful community reactions to this disclosure and therefore decide not to disclose certain information because of shared cultural identity. Training the interviewers sufficiently in asking questions about sensitive issues, keeping confidentiality, trusting and bonding, and coping with this information thus becomes key in this approach. Emphasizing the confidentiality of the shared information is crucial in every encounter in order to reassure the interviewee that the disclosed information will not be passed on to other persons in the community. In order to maintain participants’ motivation, limiting the data collection to only one interview or questionnaire per person may help to avoid attrition at follow-up.

Aside from elements influencing the feasibility and quality of research with MAR populations, challenges regarding researching the topic of SV should be addressed. First, SV needs to be defined clearly. The definition should be inclusive, that is, applicable to men, women, and transgender people of all ages, regardless of their legal status, sexual orientation, or gender identity. To achieve this objective, the acts falling under SV should be described as concrete and observable behaviours [48]. When inquiring about SV, attention should be given not only to the violent acts themselves, but also to the gender of both the victim and assailant, the context in which the violence took place, and the relation between the victim and assailant [68]. Given the broad range of types of SV MARs may have encountered, both open and closed questions regarding SV are necessary to cover the entire range of possibilities and to avoid interpretation bias [69].

When doing research on sensitive issues such as experiences of SV, it is important to consider the possibility that the study participants may experience unintended negative consequences as a result of their participation. Therefore, it may be useful to provide some follow-up support to ensure that they have not come to harm as a result of the study [70,71,72].

Developing a clean research design for SV research in MARs is quite a challenge. A balancing exercise between ethical considerations and academic standards will be key.

## 5. Conclusions

Sexual violence experiences in MARs living in Europe is widespread, yet representative studies providing a solid data base are lacking. Future research should start with a clear definition of the population and acts of SV in order to generate high-quality and comparable data. Given the necessity of acknowledging the specific experiences related to different migratory stages and motivations, mixed-method research using interviewers trained in cultural and linguistic skills should be applied to fully grasp the complex manifestations of SV in MARs.

Ultimately, the goal of prevalence studies is to have a clear view on the magnitude of the problem of SV in MARs in order to inform policymakers in their decision-making process regarding actions to improve preventive measures and the allocation of sufficient resources to care programs for both victims of SV and assailants. Although MARs are considered a specific minority group, they still are a subgroup of the general population and should thus be entitled to any general strategy to eliminate the negative consequences of SV and the victimization experience itself. In order to identify specific vulnerabilities and consequences of SV related to legal status or migratory history, comparison with the general population is necessary. Therefore, SV prevalence studies should be designed in such a way that they are applicable to representative samples from both the general population and specific subpopulations such as MARs, taking the research associated with hard-to-reach subgroups into consideration and allowing for comparisons of the findings between different populations.

## Figures and Tables

**Table 1:** Search terms. MeSH: Medical Subject Heading.

MeSH Terms	Keywords	Databases	Alerts
Prevalence; sex offences; child abuse; sexual human trafficking; rape; refugees; transients and migrants; sexual minorities	Sexual violence; rape; sexual assault; sexual abuse; child abuse; human trafficking; sexual exploitation; forced prostitution; sexual harassment; sexual slavery; attempted rape; refugees; asylum seekers; migrants; undocumented migrants; legal status; irregular; illegal; lesbian, gay, bisexual, transgender & intersex (LGBTI); conflict related sexual violence; war; European Union (EU); Europe; high-income countries; Western countries; help-seeking; help-seeking behaviour; disclosure; selective disclosure; health care; access; barriers	PubMed Google Scholar Science Direct	Crimpapers Science Direct
